# An Online Survey on Consumer Knowledge and Understanding of Added Sugars

**DOI:** 10.3390/nu9010037

**Published:** 2017-01-05

**Authors:** Mary Tierney, Alison M. Gallagher, Efstathios S. Giotis, Kristina Pentieva

**Affiliations:** 1 School of Biomedical Sciences, Ulster University, Coleraine BT52 1SA, UK; tierney_mary@hotmail.com (M.T.); am.gallagher@ulster.ac.uk (A.M.G.); 2 Faculty of Medicine, Imperial College London, London W2 1PG, UK; e.giotis@imperial.ac.uk

**Keywords:** obesity, added sugars, World Health Organisation, food product nutrition panels

## Abstract

Evidence of an association between added sugars (AS) and the risk of obesity has triggered public health bodies to develop strategies enabling consumers to manage their AS intake. The World Health Organisation (WHO) has strongly recommended a reduction of free sugars to 10% of total dietary energy (TE) and conditionally recommended a reduction to 5% TE to achieve health benefits. Despite food labelling being a policy tool of choice in many countries, there is no consensus on the mandatory addition of AS to the nutrition panel of food labels. An online survey was conducted to explore consumer ability to identify AS on food labels and to investigate consumer awareness of the WHO guidelines in relation to sugar intakes. The questionnaire was tested for participant comprehension using face-to-face interviews prior to conducting the online study. The online survey was conducted in Northern Ireland during May 2015 and was completed by a convenient sample of 445 subjects. Results showed that just 4% of respondents correctly classified 10 or more ingredients from a presented list of 13 items, while 65% of participants were unaware of the WHO guidelines for sugar intake. It may be timely to reopen dialogue on inclusion of AS on food product nutrition panels.

## 1. Introduction

Obesity is a recognised major risk factor for non-communicable diseases (NCDs) such as heart disease, stroke, cancer, chronic respiratory diseases and type 2 diabetes, which are among the leading causes of preventable morbidity. The WHO reported 1.9 billion overweight adults globally in 2014, of which 600 million were categorized as obese [1]. Diabetes currently uses approximately 10% of the National Health Service budget and this is projected to increase to 17% by 2035/36 [2]. Approximately 7 million people live with cardiovascular disease in the UK [3]. In 2012, almost 25% of adults in England were obese and a further 37% were overweight [4], and there were 11,736 hospital admissions due to obesity, more than 11 times higher than during 2001–2002 [5]. Today’s food environment tends to create a set of defaults that contribute to obesity and associated NCDs. For example, during the past 30 years the prices of healthy foods have increased at twice the rate of processed foods containing high levels of sugar and fat [6].

Sugar is increasingly being linked to the rising obesity epidemic [7]. The United States Department of Agriculture (USDA) began to use the term added sugars (AS) in 2000 to help consumers identify foods with added energy, but few additional nutrients [8]. The American Heart Association (AHA) concluded in its 2009 Scientific Statement that weight gain over the past 30 years “must be related in part to increased intake of AS” [9]. The 2015 Dietary Guidelines Advisory Committee’s (DGAC) scientific report recommended a maximum of 10 percent of daily total calories from AS, supporting changes in labelling and campaigns to increase consumer understanding of AS [10]. The WHO issued new guidelines in 2015 recommending a reduction in consumption of free sugars to 10% of total dietary energy (TE) and conditionally recommended a reduction to 5% TE for additional health benefits [11]. High intakes of free sugars are associated with poor diet quality, obesity and risk of NCDs [12]. Free sugars are defined as monosaccharides (such as glucose and fructose), disaccharides (such as sucrose) and table sugar that are added to foods and drinks by the manufacturer, cook or consumer, and sugars naturally present in honey, syrups, fruit juices and fruit juice concentrates [12]. In 2015, the Scientific Advisory Commission on Nutrition (SACN) published its report “Carbohydrates and Health” and recommended the reduction of free sugars to 5% TE or less, a recommended intake consistent with the WHO recommendations [13]. This recommendation is based on consistent evidence from cohort studies in children and adolescents that higher consumption of sugars is associated with a greater risk of dental caries, and much stronger evidence from randomised controlled trials showing that increasing or decreasing the percentage of total dietary energy as sugars leads to a corresponding increase or decrease in energy intake. Public Health England (PHE) responded to the SACN recommendations with a detailed report on tackling the crisis and continued its Change4Life campaign in January 2016 with the aim of educating the public on safe levels of sugar consumption [14]. However, a public health challenge is that no uniform definition of added and free sugars exists though generally there are many similarities in their inclusions and exclusions [15].

Nutrition labelling has emerged as a policy tool of choice for promoting healthy eating [15]. It is perceived to be a trustworthy source of information [16] and a tool for influencing consumer behaviour at the point of purchase, although its significance is debatable since consumers typically study a food label for only a few seconds [17]. The US first introduced mandatory nutrition labelling for pre-packaged food in 1990 [18] and subsequently many countries have made it mandatory [19]. There is considerable consistency in the formats of nutrition labels globally; common compulsory elements include energy, fat, saturated fat, protein, carbohydrate, sugar, and salt/sodium. AS was considered as a possible named item when nutrition labelling was first introduced in the US; however, a key argument against its inclusion was that added and naturally occurring sugars are chemically indistinguishable so would be difficult to monitor and test [20]. Despite being chemically indistinguishable, recent meta-analyses found that although there is heterogeneity in the available randomized controlled trials and cohort studies, the evidence is very consistent that the intake of AS contributes to a positive energy balance and weight gain respectively, with the effect being more marked with SSBs. Exchange of AS for other carbohydrates, however, had no effect [21]. The Food and Drug Administration (FDA) recently updated the nutrition facts panel requirements to include “added sugars”, indented under “sugars” [22].

The aim of introducing nutrition labelling was to improve consumer information and facilitate healthier eating choices, yet during the 15 years following its introduction US obesity rates continued to increase [23]. The new challenge for consumers is the management of AS intake. While the FDA has introduced labelling changes to improve transparency of sugar content, change appears less likely in Europe. The 2010 European Food Safety Authority (EFSA) report on dietary reference values for carbohydrates and dietary fibre claimed that there were insufficient data to set an upper limit for (added) sugar intake as the evidence is inconsistent for the relationship between consumption of sugary foods and dental caries and weight gain [24]. Under current EU labelling regulations [25] consumers can only determine the AS content by looking at the ingredients listing, which is challenging as the food industry has been accused of disguising AS under unfamiliar names [26]. Despite the strong rationale behind consumer education and prevention strategies outlined above, there has been limited research exploring consumers’ understanding of dietary sugars [27,28]. The aims of this study were two-fold: (1) to explore consumer ability to identify AS, essential to managing intake and (2) to investigate consumer awareness of the WHO guidelines in relation to dietary sugar intakes.

## 2. Materials and Methods

### 2.1. Survey Procedures

The research was conducted as an anonymous online survey via the online platform SurveyMonkey (www.surveymonkey.com). The Ethical Filter Committee at Ulster University reviewed the survey and granted approval to proceed (FCBMS-15-006). Informed consent was obtained from participants through a few screening questions before the completion of the main questionnaire (Supplementary Materials File 1). The survey was completed by participants during May 2015.

### 2.2. Participants

Participants were recruited through the Ulster University staff and students mailing list. Screening questions were used to ensure that participants were 18 years or over and were resident in the UK. Participants were requested to invite family, friends and/or colleagues to participate by forwarding the online survey link. The opportunity to win one of three commercial vouchers (£25) was used as an incentive.

### 2.3. Questionnaire

Prior to conducting the online survey, the questionnaire was tested for participant comprehension using face-to-face interviews with a small number of people (*n* = 8), again a convenient sample. The group included households with and without children and included a mix of ages. Following each interview, the questions were discussed and minor wording amendments were made as the interviews progressed. Demographic profiling questions included gender, age, highest level of education and presence of children under 18 in the household; age data were collected using categories that align with the UK Census categories [29]. Label use, including frequency of looking at labels, items of interest on the label and perception of the importance of various macronutrients, was explored. Two further closed-ended questions explored awareness of the WHO guidelines on free sugars (Supplementary Materials File 2) and perceived ease of implementing the guidelines using current label information. Respondent ability to classify sugars and artificial sweeteners was investigated by asking them to categorise a list of 13 items as presented in Table 1. Two open-ended questions were included to enable respondents to provide feedback on their approach to managing sugar intake. The questionnaire is presented as Supplementary Materials File 1.

### 2.4. Statistical Analysis

The sample size for the current study was estimated by using the proportion of correct responses obtained in a survey investigating the knowledge of the consumers for the presence and the relative content of added sugar in food products [28]. It was estimated that a sample size of 500 participants would be required in order to obtain 5% correct responses for the identification of AS with a power of 80% at α = 0.05.

SPSS software (version 22.0) was applied for all statistical analyses. Descriptive statistics and crosstabs were used for analysis of participants’ characteristics. Open-ended questions were coded in order to quantify the responses. Chi-square tests were applied to test for differences between different categories and where cell values were low Fishers Exact test was applied.

A scoring system was applied for assessment of participants’ knowledge in relation to classification of added sugars as follows: respondents were given a score of 1 for each correctly classified ingredient, with a maximum possible score of 13; a higher score indicated better participant understanding of AS. One-way ANOVA was used to compare scores across demographic and participant characteristics categories followed by Dunnett post-hoc test. A *p* < 0.05 was used to determine significance.

## 3. Results

### 3.1. Respondent Profile

Of the 502 completed online questionnaires, 445 were considered eligible with all the questions answered and 47 were rejected because participants dropped out part way through the survey. The average time taken to complete the survey was 8 min.

Demographic and other characteristics of the sample population are presented in Table 2.

The majority (79%) of the participants were 18–54 years old. Of those who completed the questionnaire significantly higher numbers were female and two-thirds (65%) were educated to a degree level or above. Less than half had children under 18 years living at home. The majority of participants claimed to be interested or very interested in nutrition. Self-reported use of food labels was high with 84% claiming that they at least sometimes look at labels (*p* < 0.001). Label usage was significantly higher for females than males (*p* = 0.043), among those with an interest in nutrition (*p* < 0.001) and those who find the traffic light system helpful (*p* < 0.001). There were no differences by age (*p* = 0.073), presence of children in the household (*p* = 0.333) or education (*p* = 0.778).

### 3.2. Current Use of the Nutrition Panel 

**Figure 1:**
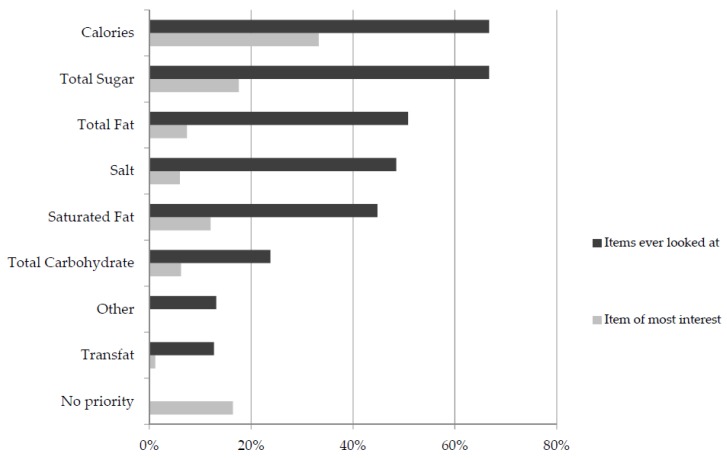
Nutrition panel items looked at by consumers and item of most interest, *n* = 433.

Participants were asked which items on the nutrition panel they usually look at (Figure 1) and reported that calories, total sugar, total fat, salt and saturated fat were most frequently looked at. When asked to prioritise the item of most interest energy was most likely to be selected, followed by total sugar and saturated fat (Figure 1). Almost one fifth of respondents indicated no priority with respect to looking at energy/macronutrients on the label. There were no significant differences by gender (*p* = 0.129), age (*p* = 0.220) or education (*p* = 0.411).

Participants were also asked to nominate the item they believe is most important to consider in order to stay healthy; saturated fat (28%), sugar (23%), calories (12%) and fat (11%) were most likely to be selected and no difference between responses were observed for gender (*p* = 0.737), age (*p* = 0.284) or education (*p* = 0.366). In contrast relationships were observed between item “most important in order to stay healthy” and “interest in food and nutrition” (*p* = 0.014) and “frequency of label use” (*p* = 0.011). Despite respondents stating that calories were “of most interest”, they considered that limiting the intake of saturated fat (24%), total fat (13%) and sugar (12%) were “most important in order to stay healthy”.

The traffic light front-of-pack (FOP) system was rated as helpful by the majority of respondents (81%). There were no differences by gender (*p* = 0.323), presence of children in the household (*p* = 0.05) or level of education (*p* = 0.447). Younger participants, aged 18–24 years, (*p* = 0.031), those who are interested in food and nutrition (*p* = 0.002) and frequent users of food labels (*p* < 0.001) were more likely to believe it is helpful.

### 3.3. Awareness of the WHO Recommendation for Sugar Reduction

The majority (65%) of participants reported no knowledge of the revised WHO guidelines. Awareness did not differ by gender (Males vs. Females: 38% vs. 27%, *p* = 0.061), education (High school vs. College vs. Degree vs. Post grad: 33% vs. 39% vs. 29% vs. 40%, *p* = 0.296), presence of children in the household (Yes vs. No: 32% vs. 37%, *p* = 0.285) or frequency of label use (Always vs. Sometimes vs. Hardly ever/Never: 40% vs. 36% vs. 33%, *p* = 0.111) whereas differences by age (18–24 years vs. 25–34 years vs. 35–44 years vs. 45–54 years vs. 55–64 years vs. 65+ years: 27% vs. 25% vs. 34% vs. 36% vs. 42% vs. 72%, *p* < 0.001) and interest in nutrition (Very interested vs. Interested vs. Not very/Not at all interested: 46% vs. 32% vs. 24%, *p* = 0.004) were observed.

Participants were presented with a summary of the WHO guidelines and asked to rate the ease of implementing the recommendations based on current labelling. Only 3% reporting that it would be “very easy”, while a majority believed it would “not be very easy” (45%) or “not easy at all” (25%). In terms of the % selecting “very easy”, there were no significant differences by gender (Males vs. Females: 4% vs. 2%, *p* = 0.642), awareness of the WHO guidelines (Yes vs. No: 4% vs. 2%, *p* = 0.624), presence of children in the household (Yes vs. No: 4% vs. 3%, *p* = 0.403), or education (High school vs. College vs. Degree vs. Post grad: 5% vs. 3% vs. 3% vs. 3%, *p* = 0.853). Significant differences were identified by frequency of label usage (Always vs. Sometimes vs. Hardly ever/Never: 8% vs. 1% vs. 1%: *p* < 0.001), interest in nutrition (Very interested vs. Interested vs. Not very/Not at all interested: 7% vs. 2% vs. 0%, *p* < 0.001), perception of helpfulness of the traffic light system (Very helpful vs. Somewhat helpful vs. Not very/Not at all helpful: 5% vs. 3% vs. 2%, *p* < 0.001) and age (18–24 years vs. 25–34 years vs. 35–44 years vs. 45–54 years vs. 55–64 years vs. 65+ years: 6% vs. 3% vs. 1% vs. 3% vs. 3% vs. 3%, *p* = 0.010).

### 3.4. Ability to Correctly Classify Dietary Sugars and Sweeteners

**Figure 2:**
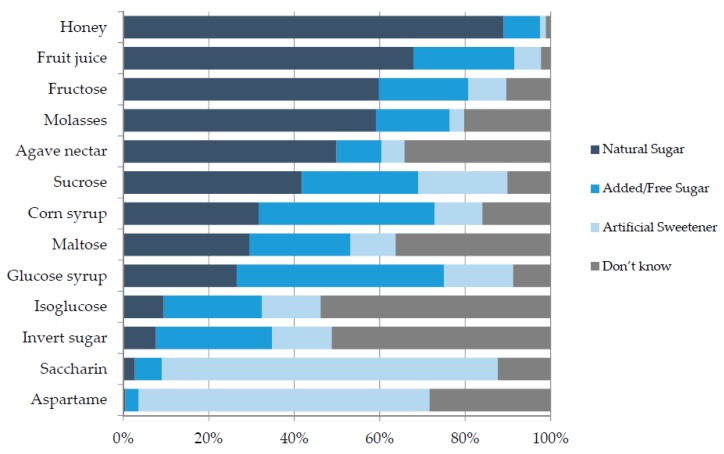
Consumer classification of sugars and sweeteners, *n* = 443.

An important aim of the research was to explore participant ability to identify AS on food labels. Participants were asked to classify each of 13 commonly used food ingredients as a natural sugar, an AS or an artificial sweetener (Figure 2).

In the present study, all ingredients presented were categorised as either AS or artificial sweeteners in accordance with WHO guidelines [12], yet many participants misclassified these as natural sugars. For example, honey, when used as an ingredient, was incorrectly classified as a natural sugar by 89% while fruit juice was incorrectly classified as a natural sugar by 69%. Commonly used ingredients such as invert sugar and isoglucose could not be classified by half of the participants. In contrast, saccharin and aspartame were correctly classified as artificial sweeteners by the majority (60%–80%) of respondents. 

Table 3 presents mean consumer scores by demographic groups and other profiling variables. The mean consumer score across the total sample was 4.2 (SD 2.7) out of a possible maximum score of 13 (i.e., on average only four of 13 ingredients were correctly classified by respondents). Just 4% of respondents correctly classified 10 or more ingredients while almost half could only correctly identify three ingredients or fewer. 

Determinants with a positive impact of achieving a high score were frequency of label usage (*p* = 0.039), attitude to the traffic light system (*p* = 0.004), interest in food and nutrition (*p* < 0.001) and education (*p* = 0.027). Differences by gender (*p* = 0.403), age (*p* = 0.893) and perception of ease of implementing the WHO guidelines (*p* = 0.062) were not significant. 

When respondents were asked if there were items on the list that they would actively avoid, the artificial sweeteners aspartame (48%) and saccharin (44%) were most likely to be selected. Other items actively avoided included corn syrup (29%), glucose syrup (25%), sucrose (23%) and invert sugar (21%). Almost a third (29%) would not avoid any of the listed ingredients. 

Participants were also asked to classify sugars naturally present in fruit and milk. In the case of “sugars in fresh fruit and vegetables” 97% classified them as natural sugars and 4% as added sugars or free sugar. “Sugars present in milk (lactose)” were classified as natural sugar by 83% while 13% believed that the sugars in milk are added or free sugars with 4% selecting “don’t know”.

### 3.5. Consumer Approach to Managing Sugar Intake 

Responses to the open-ended question which explored consumers’ approach to managing their sugar intake were classified into three major themes, which accounted for three-quarters of participants’ responses. They considered that avoiding processed and pre-packaged foods (27%) was important, as was avoiding obviously sugary foods such as cakes, biscuits, fizzy drinks and fruit juices (27%). Use of current labels (21%) was also viewed as an important aid when it comes to managing sugar intake.

In terms of what consumers would find most helpful in managing sugar intake they reported that labelling needed to be improved (25%); they wanted larger font, less information, realistic portion sizes. Colour coding of sugar (15%) above the recommended level was also requested, while 13% believed that the current labels were the best aid. Almost a tenth (8%) suggested that specifying sugar content in teaspoons would be helpful.

## 4. Discussion

To our knowledge this is the first study to specifically assess consumer understanding of AS ingredients, though work has been conducted on related topics [27,28]. The present study observed that awareness of the WHO guidelines [11] for AS consumption was low, even among highly-educated consumers, those with an interest in food and nutrition and those who frequently consult food labels. Approximately two-thirds (65%) of participants were unaware of the WHO revised guidelines and awareness was significantly lower amongst younger people and those who were not interested in nutrition. Only 3% of participants believed that it would be “very” easy to implement the guidelines using current labels, while 70% felt it would not be very easy or not easy at all. Only 4% of participants correctly classified 10 or more ingredients from a presented list of 13 AS items with overall ability to correctly identify AS being poor even for consumers who claimed to be interested in nutrition and/or always look at labels. In the present study most respondents viewed traffic light FOP graphics favourably for alerting consumers to AS content of foods and respondents supported simplifying product labels. 

Low consumer awareness of the WHO guidelines for AS consumption is just part of the issue; a greater challenge for public health bodies is in supporting and facilitating targets for achieving reduced sugar consumption. Having presented participants with a summary of the guidelines it is concerning that only 3% of participants believed that it would be “very” easy to implement the guidelines using current labels. Food labels are a tool for providing information to consumers as confirmed by high usage of participants in the present study. The high level of label usage is consistent with other studies that have found self-reported use of nutrition labels to be prevalent [15] and typically above 50% [30]. Label use tends to be higher among those with higher levels of education [31] therefore it could be hypothesised that label use may be lower amongst a broader sample of the general population. Nevertheless, nutrition labels are largely considered an important source of information to consumers. 

However, when it comes to AS consumers struggle as AS are often hidden within the food labels by the use of unfamiliar names [26]. In the present study, ingredients such as honey, fruit juice or fructose, which under the WHO guidelines [11] are categorised as AS, were incorrectly classified as natural sugars by large proportions (60%–89%) of participants. Significantly, this holds true even for frequent users of labels, for those who believed that it would be easy to implement the guidelines and for respondents with an interest in nutrition. Maybe this misclassification is just a result of inappropriate usage of “natural sugars” as in lay-man terms it is associated with those sugars which are normal ingredients of non-processed foods. The present study highlights that educating consumers on how to identify AS may be important. Buckton et al. [32] found that perceptions of messages linking health and diet were influenced by the terminology used and recommended tailored approaches to public health campaigns to meet the needs of different groups. Creating uniform definitions for added, free and natural sugars may be important as they don’t exist today [33].

Respondents were most likely to correctly classify the artificial sweeteners saccharin and aspartame. Interestingly, respondents in the present study were also more likely to state that they actively avoid artificial sweeteners than any of the AS examined. Despite extensive safety evaluation of artificial sweeteners, consumers remain sceptical in relation to their use [34].

When given the opportunity to express their own views on how they would manage sugar intake, if they were trying to reduce its consumption, the most frequent response was that they believed that avoiding processed foods and sugary food and drink is important. This suggests that sugars “hidden” in savoury and less obviously sweet foods may be not considered as participants believe that reducing consumption of sugary foods and drinks is sufficient. The WHO acknowledged that sugar is often “hidden” in savoury foods [11]. Additionally, respondents in the present study reported that it can be difficult to implement any self-restraint in an environment where processed sugary foods are readily available and more affordable. There was a desire for simpler food labelling; consumers were concerned that portion sizes can be misleading and that it can be difficult for the average consumer to calculate from per 100 g data to per portion. This is consistent with research in New Zealand, which found that understanding of labels was problematic for many consumers [35]. Campos et al. [30] recommended the exploration of new formats of food labels with different information content in order to improve comprehension and accessibility. Almost a tenth of respondents in the current study suggested that the declaration of sugar in teaspoons would be helpful. A benefit of this approach could be its simplicity and alignment with the language of the WHO guidelines [11]. The traffic light FOP graphics were viewed favourably; these findings align with other studies where consumers reported that the use of graphics helped them to identify healthier food options [36,37,38]. Gorton [35] found that traffic light food labels were understood across consumer groups with different ethnicity, income, and education levels as well as amongst irregular label users. Declaring total AS using the traffic light FOP and/or adding it as a mandatory item on the nutrition panel could support consumers in managing their sugar intake with clearly defined thresholds for low medium and high sugar content [39]. FOP graphics may be more effective than educating consumers to identify specific ingredients.

Amongst respondents who reported to regularly look at food labels, energy (calories) and total sugar were the items most commonly considered, followed by fat, salt and saturated fats; this overall pattern is consistent with findings by Grunert et al. [40]. Respondents in the current study prioritized energy (calories) as the item of most interest, followed by total sugar. Educating consumers to pay particular attention to the sugar content appears to be important in an environment where sugar intake is increasingly associated with a number of risk factors. In terms of what participants believe to be most important to watch in order to stay healthy, saturated fat and total sugar were most likely to be selected in the present study. It is not surprising that consumers still rate saturated fat as important given that health messages have focused on this macronutrient for decades [7], further emphasising the value of consumer education. The fact that sugar was ranked second may indicate that the message in relation to managing sugar intake is getting through to consumers. 

The present study was based on a convenient sample of consumers and was therefore not representative of the general population. A further limitation of the study was the overrepresentation of female respondents (77%), under representation of over 65 s compared to population level [29] and the inclusion of a higher proportion of degree educated (or higher) consumers. Interestingly, despite the group being relatively highly educated, participants struggled to correctly classify AS. Additional limitations relate to the survey being conducted online and through the University database, which may fail to reach minority groups both in terms of education and ethnicity. Thus, it is possible that these results present a “best-case scenario”, as less educated consumers may have an even poorer understanding of sugar issues and may be more at risk of increased exposure to the obesogenic environment. Further, previous research has indicated that lower compared with higher socio-economic groups were less knowledgeable on nutrition issues [41]. A follow-up study with a representative sample of the general population is thus advised.

## 5. Conclusions

In conclusion, the present study highlights for the first time that even relatively well-educated participants struggled to understand food content of AS using current nutrition labels. Without the requirement to include total AS on labels, consumers are reliant on the ingredients listing and this study has demonstrated that the majority cannot correctly identify AS. Awareness of the WHO guidelines [11] was low and may be lower amongst a random sample of the general population. While the WHO guidelines are aimed at practitioners, effective means of translating and communicating them to the consumer need to be found. Governments, policy makers and public health bodies need to examine how they can more effectively support consumers in decreasing their sugar intake. It raises the question as to whether the food and catering industries should also play an active role in supporting consumers to decrease sugar consumption. Declaring total AS on the label, whether on the nutrition panel or by traffic light graphics, has the potential to have wide reach of consumers considering the availability of the tool. The inclusion of food labelling in public health strategies to tackle obesity warrants consideration. 

## Figures and Tables

**Table 1:** Sugars and artificial sweeteners that were classified by participants.

Sugars	Artificial Sweeteners
Agave Nectar	Aspartame
Corn syrup	Saccharin
Fructose	-
Fruit juice	-
Glucose	-
Honey	-
Invert sugar	-
Isoglucose	-
Maltose	-
Molasses	-
Sucrose	-

**Table 2:** General characteristics of participants.

	*n*	%	*p* Value ^1^
Gender			<0.001
Female	338	77	
Male	102	23	
Age (years)			<0.001
18–24	79	18	
25–34	69	16	
35–44	104	23	
45–54	97	22	
55–64	62	14	
65–74	19	4	
75+	10	2	
Prefer not to say	3	1	
Education			<0.001
High school	40	9	
College	108	24	
Degree	154	35	
Post grad	132	30	
Prefer not to say	9	2	
Children Under 18 in Household			<0.001
Yes	167	38	
No	273	62	
Frequency of looking at Labels			<0.001
Always	111	24	
Sometimes	308	61	
Hardly ever	66	13	
Never	15	3	
Helpfulness of traffic light system			<0.001
Very helpful	153	34	
Somewhat helpful	210	47	
Not very helpful	31	7	
Not helpful at all	21	5	
Don’t know	30	7	
Interest in food & nutrition			<0.001
Very interested	138	31	
Interested	257	58	
Not very interested	46	10	
Not interested at all	3	1	
Don’t know	1	0	

^1^ Data were analysed using Chi Square test; *n* = 445.

**Table 3:** Consumer score of correctly classified added sugars according to demographic and other general characteristics.

	Mean	Std. Deviation	*p* Value ^1^
Total	4.2	2.7	
*Frequency of looking at labels*			0.039
Always	4.6 ^a^	2.9	
Sometimes	4.2 ^a^	2.6	
Hardly ever/Never	3.4 ^b^	2.4	
*Helpfulness of traffic light system*			0.004
Very helpful	4.1 ^a^	2.5	
Somewhat helpful	4.2 ^a^	2.6	
Not very/Not at all helpful	5.1 ^b^	3.3	
Don’t know	2.9 ^c^	2.0	
*Interest in food & nutrition*			<0.001
Very interested	5.2 ^a^	2.8	
Interested	3.7 ^b^	2.5	
Not very/not at all interested	3.7 ^b^	2.4	
*Highest level of education*			0.027
High school/College	3.6 ^a^	2.4	
Degree	4.3 ^b^	2.8	
Post grad	4.6 ^b^	2.7	
*Gender*			0.403
Male	4.0	2.8	
Female	4.2	2.6	
*Age*			0.893
18–24	4.1	2.9	
25–34	3.7	2.4	
35–44	4.4	2.7	
45–54	4.3	2.7	
55–64	4.2	2.6	
65–74	4.1	2.8	
75+	4.0	2.4	

^1^ Data were analysed using One-way ANOVA followed by Dunnett post-hoc test. Means with different superscript letters indicate significant differences; *n* = 445.

## Supplementary Materials

The following are available online at http://www.mdpi.com/2072-6643/9/1/37/s1, File S1: Questionnaire, File S2: WHO Press Release.

## Abbreviations

The following abbreviations have been used in this manuscript:

AHA	American Heart Association
AS	Added Sugars
BHF	British Heart Foundation
DGAC	Dietary Guidelines Advisory Committee
EFSA	European Food Safety Authority
EUFIC	European Food Information Council
FDA	US Food and Drug Administration
FOP	Front-of-pack
HSCIC	Health and Social Care Information Centre
NCD	Non-Communicable Disease
NHS	National Health Service
PHE	Public Health England
SACN	Scientific Advisory Commission on Nutrition
TE	Total Dietary Energy
USDA	United States Department of Agriculture
WHO	World Health Organisation
