# Supplementary material for: An Online Survey on Consumer Knowledge and Understanding of Added Sugars
Source: Nutrients. 2017 Jan 5;9(1):37. doi: 10.3390/nu9010037 (PMC5295081; doi:10.3390/nu9010037)
Supplement: Supplementary file 1 [file nutrients-09-00037-s001.docx]

Supplementary Materials: An Online Survey on Consumer Knowledge and Understanding of
Added Sugars

Mary Tierney, Alison M. Gallagher, Efstathios S. Giotis and Kristina Pentieva

**File 1—Questionnaire**

We are conducting research on labelling of foods and would be very grateful if you can take some time to answer these questions. We estimate that it will take around 6–8 min.

Screening Questions:

Respondents must answer ‘yes’ to all screening questions before progressing to Q1.

1. I confirm that I have been given and have read and understood the information sheet for the above study and have asked and received answers to any questions raised.
2. I understand that my participation is voluntary and that I am free to withdraw at any time without giving a reason and without my rights being affected in any way.
3. I understand that the researchers will hold all information and data collected securely and in confidence and that all efforts will be made to ensure that I cannot be identified as a participant in the study—I agree to take part in the above study.
4. I am over 18 years.
5. I live in the UK.

**Quantitative questionnaire**

1. How often do you look at nutrition labels on food when you are purchasing?
2. Always
3. Sometimes
4. Hardly ever
5. Never—Go to Q4
6. Which items on the label do you look at? *Tick all that apply*
7. Calories
8. Total Fat
9. Saturated Fat
10. Trans fat
11. Total carbohydrate
12. Total Sugar
13. Salt
14. Other (Please specify)
15. Is there one item in particular that you are interested in? *Tick one only*
16. Calories
17. Total Fat
18. Saturated Fat
19. Trans fat
20. Total carbohydrate
21. Total Sugar
22. Salt
23. No priority
24. Which one of the following do you believe is the most important to watch in order to stay healthy? *Tick one only*
25. Calories
26. Carbohydrates
27. Sugar
28. Fat
29. Saturated fat
30. Protein
31. Alcohol
32. Salt
33. Pre-packaged food labels contain a list of ingredients. If you saw the following items listed how would you classify them? Please categorise each one as natural sugar, added/free sugar or an artificial sweetener...

*Note: Added/Free sugars are those that are added to foods during manufacturing/cooking.*

|  | **Natural Sugar** | **Added/Free Sugar** | **Artificial Sweetener** | **Don’t Know** |
| --- | --- | --- | --- | --- |
| Glucose |  |  |  |  |
| Sucrose |  |  |  |  |
| Saccharin |  |  |  |  |
| Fructose |  |  |  |  |
| Maltose |  |  |  |  |
| Honey |  |  |  |  |
| Agave nectar |  |  |  |  |
| Molasses |  |  |  |  |
| Fruit juice |  |  |  |  |
| Corn syrup |  |  |  |  |
| Aspartame |  |  |  |  |
| Invert sugar |  |  |  |  |
| Isoglucose |  |  |  |  |

1. And what about the following items—how would you classify them?

|  | **Natural Sugar** | **Added/Free Sugar** | **Don’t Know** |
| --- | --- | --- | --- |
| Sugars present in milk (Lactose) |  |  |  |
| Sugars in fresh fruit and vegetables |  |  |  |

1. Which, if any, would you actively avoid? *Tick all that apply*
2. Glucose
3. Sucrose
4. Saccharin
5. Fructose
6. Maltose
7. Honey
8. Agave nectar
9. Molasses
10. Corn syrup
11. Fruit juice
12. Aspartame
13. Invert sugar
14. Isoglucose
15. Sugars present in milk (Lactose)
16. Sugars in fresh fruit and vegetables
17. None
18. Have you heard of the World Health Organisation (WHO) recommendation for the reduction of added sugar to 5% of daily intake for additional health benefits?
19. Yes
20. No
21. The WHO published a new guideline in March 2015 recommending that sugar should make up no more than 10% of daily energy intake. It further recommended a reduction to 5% for additional health benefits. 5% is the equivalent of 9 teaspoons for men and 6 teaspoons for women.

The sugars in question are Added Sugars, i.e., sugars that are added to foods during processing or cooking and those naturally present in honey, fruit juice and syrup.

Based on current labelling how easy would it be for you to monitor and plan total sugar intake? Would you say:

1. Very easy
2. Fairly easy
3. Not very easy
4. Not easy at all
5. Don’t know
6. How do you think you would manage your sugar intake if you were trying to reduce consumption? I’m interested in the type of approach you might take.

|  |
| --- |
|  |

1. Thinking about when you go food shopping what would be the most important aid to you in understanding the sugar content of food?

|  |
| --- |
|  |

1. How helpful do you find the Traffic Light system where food elements such as Fat, Carbohydrate, Sugar and Salt are coded red, amber or green depending on the levels in the food?
2. Very helpful
3. Somewhat helpful
4. Not very helpful
5. Not helpful at all
6. Don’t know
7. How interested are you in food and nutrition? Would you say
8. Very interested
9. Interested
10. Not very interested
11. Not interested at all
12. Don’t know

**Demographics**

1. Gender
2. Male
3. Female
4. Age
5. 18–24
6. 25–34
7. 35–44
8. 45–54
9. 55–64
10. 65–74
11. 75+
12. Prefer not to say
13. What is the highest level of education that you have attained?
14. High school
15. College
16. Degree
17. Post grad
18. Prefer not to say
19. Are there children aged under 18 living in your household?
20. Yes
21. No

File 2—WHO Press Release

WHO calls on countries to reduce sugars intake among adults and children

*Press release*

4 March 2015 ¦ GENEVA—A new WHO guideline recommends adults and children reduce their daily intake of free sugars to less than 10% of their total energy intake. A further reduction to below 5% or roughly 25 grams (6 teaspoons) per day would provide additional health benefits.

Guideline on sugars intake for adult and children

Free sugars refer to monosaccharides (such as glucose, fructose) and disaccharides (such as sucrose or table sugar) added to foods and drinks by the manufacturer, cook or consumer, and sugars naturally present in honey, syrups, fruit juices and fruit juice concentrates.

“We have solid evidence that keeping intake of free sugars to less than 10% of total energy intake reduces the risk of overweight, obesity and tooth decay,” says Dr Francesco Branca, Director of WHO’s Department of Nutrition for Health and Development. “Making policy changes to support this will be key if countries are to live up to their commitments to reduce the burden of noncommunicable diseases.”

The WHO guideline does not refer to the sugars in fresh fruits and vegetables, and sugars naturally present in milk, because there is no reported evidence of adverse effects of consuming these sugars.

Much of the sugars consumed today are “hidden” in processed foods that are not usually seen as sweets. For example, 1 tablespoon of ketchup contains around 4 grams (around 1 teaspoon) of free sugars. A single can of sugar-sweetened soda contains up to 40 grams (around 10 teaspoons) of free sugars.

Worldwide intake of free sugars varies by age, setting and country. In Europe, intake in adults ranges from about 7%–8% of total energy intake in countries like Hungary and Norway, to 16%–17% in countries like Spain and the United Kingdom. Intake is much higher among children, ranging from about 12% in countries like Denmark, Slovenia and Sweden, to nearly 25% in Portugal. There are also rural/urban differences. In rural communities in South Africa intake is 7.5%, while in the urban population it is 10.3%.

Reducing sugars intake to less than 10% of total energy: a strong recommendation

The recommendations are based on analysis of the latest scientific evidence. This evidence shows, first, that adults who consume less sugars have lower body weight and, second, that increasing the amount of sugars in the diet is associated with a weight increase. In addition, research shows that children with the highest intakes of sugar-sweetened drinks are more likely to be overweight or obese than children with a low intake of sugar-sweetened drinks.

The recommendation is further supported by evidence showing higher rates of dental caries (commonly referred to as tooth decay) when the intake of free sugars is above 10% of total energy intake compared with an intake of free sugars below 10% of total energy intake.

Based on the quality of supporting evidence, these recommendations are ranked by WHO as “strong”. This means they can be adopted as policy in most situations.

Further reduction to less than 5% of total energy intake: a conditional recommendation

Given the nature of existing studies, the recommendation of reducing intake of free sugars to below 5% of total energy is presented as “conditional” in the WHO system for issuing evidence-based guidance.

Few epidemiological studies have been undertaken in populations with a low sugars intake. Only three national population-wide studies allow a comparison of dental caries with sugars intakes of less than 5% of total energy intake versus more than 5% but less than 10% of total energy intake.

These population-based ecological studies were conducted during a period when sugars availability dropped dramatically from 15 kg per person per year before the Second World War to a low of 0.2 kg per person per year in 1946. This “natural experiment”, which demonstrated a reduction in dental caries, provides the basis for the recommendation that reducing the intake of free sugars below 5% of total energy intake would provide additional health benefits in the form of reduced dental caries.

WHO issues conditional recommendations even when the quality of evidence may not be strong on issues of public health importance. A conditional recommendation is one where the desirable effects of adhering to the recommendation probably outweigh the undesirable effects but these trade-offs need to be clarified; therefore, stakeholder dialogue and consultations are needed before the recommendation is implemented as policy.

Updating the guideline on free sugars intake is part of WHO’s ongoing efforts to update existing dietary goals to prevent NCDs. The sugars guidelines should be used in conjunction with other nutrient guidelines and dietary goals, in particular those related to fats and fatty acids, including saturated fat and trans-fat.

In March 2014, WHO opened a public consultation on the then draft sugars guideline to seek inputs from all stakeholders. More than 170 comments were received from representatives of government agencies, United Nations agencies, nongovernmental organizations, industries and academic institutions as well as other interested individuals. An expert peer review process was also undertaken in 2014. The final guideline was prepared taking into account comments received from the public consultation and expert peer review.

Countries can translate the recommendations into food-based dietary guidelines that consider locally available food and customs. Additionally, some countries are implementing other public health interventions to reduce free sugars intake. These include nutrition labelling of food products, restricting marketing to children of food and non-alcoholic drinks that are high in free sugars, fiscal policies targeting foods and beverages high in free sugars, and dialogue with food manufacturers to reduce free sugars in processed foods.
